# Investigating orbital foreign device-associated malignancies: a scoping review

**DOI:** 10.1186/s12885-024-13422-z

**Published:** 2025-01-28

**Authors:** Jeong Hyun Ha, Jagmeet S. Arora, Jamasb J. Sayadi, Nada R. Khattab, Shannon D. Francis, Thomas M. Johnstone, Yeonji Jang, Gordon K. Lee

**Affiliations:** 1 Interdisciplinary Program of Medical Informatics, Seoul National University College of Medicine, Seoul, South Korea; 2 Plastic and Reconstructive Surgery, CHA Gangnam Medical Center, CHA University School of Medicine, Pocheon, South Korea; 3 School of Medicine, University of California, Irvine, CA USA; 4 Department of Neurosurgery, Stanford University, Stanford, CA USA; 5 Department of Plastic Surgery, The Ohio State University, 915 Olentangy River Road, Columbus, OH USA; 6 Stanford University School of Medicine, Stanford, CA USA; 7 Department of Ophthalmology, Uijeongbu Eulji Medical Center and Eulji University, Uijeongbu, South Korea; 8 Department of Plastic Surgery, University of California, Irvine, CA USA; 9 Department of Plastic and Reconstructive Surgery, Cha Gangnam Medical Center, Seoul, South Korea; 10 Department of Plastic Surgery, School of Medicine, University of California, Irvine, Irvine, CA USA

**Keywords:** Orbital prosthetic device, Orbital foreign device, Conjunctival carcinoma, Sarcoma, Ocular malignancy

## Abstract

**Background:**

While prosthesis-associated malignancies have been acknowledged, awareness among surgeons and patients in the ophthalmologic field remains limited, despite the frequent occurrence of prosthesis-related surgeries. We aim to address this gap through a scoping review of malignancies following ophthalmologic surgeries involving various foreign device/prosthesis/implants.

**Methods:**

Following PRISMA guidelines, we conducted a review using PubMed and Embase for studies on cancer and ophthalmic prostheses/implants. The final selection of articles for the ophthalmology aspect underwent rigorous investigation.

**Results:**

We analyzed 30 studies, identifying 41 cases of malignancies following interventions involving orbital foreign devices. Foreign devices linked to malignancies included scleral shells, orbital implants, scleral buckles, encircling bands, and gold plates. Ocular surface squamous neoplasm was most common, with 29 cases. Other malignancies observed were pleomorphic sarcoma, synovial sarcoma, marginal zone B-cell lymphoma, sebaceous carcinoma, malignant melanoma, adenocarcinoma, and metastatic lung adenocarcinoma. These malignancies varied in characteristics and demographics, with some showing metastasis or local invasion. The median time to tumor development was 40 years.

**Conclusions:**

This study highlights a spectrum of malignancies following orbital foreign device procedures and suggests a potential association between different types of orbital foreign devices and malignancies. Future studies, including well-designed epidemiologic studies and scientific experiments, are warranted to elucidate the causal relationship.

**Supplementary Information:**

The online version contains supplementary material available at 10.1186/s12885-024-13422-z.

## Introduction

Various prosthesis are routinely utilized in ophthalmologic procedures including intraocular pressure lowering surgery for glaucoma, buckling or encircling for retinal detachment repair, and cataract surgery. Although less common, the insertion of orbital implants following enucleation remains a vital procedure for orbital volume maintenance. Scleral shells, also called ocular prosthesis, are fitted over the sclera to restore natural appearance. Despite the widespread use of these foreign materials, there is limited literature reporting malignancies occurring after foreign body procedures.

The potential for prosthesis-associated malignancies has been recognized since Oppenheimer reported carcinogenesis following foreign body implantation in animal model in 1958 [1]. However, comprehensive studies investigating malignancy development following prosthesis-related procedures in ophthalmology are notably absent. While isolated case reports, often accompanied by literature reviews, have documented occurrences of squamous cell carcinoma (SCC) following the prolonged use of scleral shells [2–8], there remains a significant gap in the literature regarding a comprehensive review of prosthesis-related malignancies within the ophthalmologic field. These sporadic reports provide valuable insights into potential risks associated with foreign device in ophthalmologic surgeries. However, a thorough and systematic review encompassing all orbital foreign device-related malignancies is notably absent from the current literature.

Moreover, despite the common incidence of prosthesis-related surgeries in ophthalmologic field, awareness of the possibility of prosthesis-associated malignancies among surgeons and patients is limited. It is essential for clinicians to remain vigilant and consider the possibility of malignancy during patient surveillance, as early detection can significantly impact patient outcomes.

The recent emergence of breast implant-associated anaplastic large cell lymphoma (BIA-ALCL) [9, 10] has underscored the importance of vigilance regarding implant-associated malignancies. While BIA-ALCL initially went unnoticed, its increasing incidence and awareness has prompted changes in the trends of breast implant surgeries. Considering the substantially higher incidence of breast implant surgeries compared to other fields, it is conceivable that other prosthesis-associated malignancies may be underreported or overlooked in ophthalmology due to their lower incidence rates.

Our study aimed to comprehensively review malignancies occurring after ophthalmic prostheses, systematically map research conducted in this area, and identify any existing gaps in knowledge. By providing insights into orbital foreign device-associated malignancies, our study aims to raise awareness and advise caution where necessary. Increased awareness of potential prosthesis-associated malignancies can guide the appropriate diagnosis and treatment when necessary, significantly impacting patient safety and clinical practice.

## Materials and methods

### Design

This scoping review used the PRISMA Extension for Scoping Reviews (PRISMA-ScR) as a guideline for content. As a review of existing literature, the study did not require Institutional Review Board (IRB) approval.

### Objectives

This review sought to comprehensively identify and summarize the existing literature in which malignancies occurred after ophthalmic prostheses. Studies of human subjects developing de novo malignancies after the use of a foreign device or prosthesis were included. Articles describing metastatic tumors occurring adjacent to the site of implantation were also included.

### Search strategy

The search strategy was developed iteratively with input from an experienced medical librarian. The search was developed in PubMed and designed to be comprehensive. It was translated to work optimally in Embase and Cochrane CENTRAL. It is not possible to specify a time sequence or causation vs. correlations in a database search. The search identified a large body of biomedical literature in which both cancer and prostheses/implants/devices were subjects. Articles concerning malignancies following implantation or use of a foreign device or prosthesis in humans were eligible for this review. Specific details of the search strategy can be found in Appendix S1.

### Inclusion and exclusion criteria

Given the rarity of malignancies reported in association with prostheses, implants, and devices, our research includes articles, abstracts, and conference proceedings concerning malignancies following implantation or the use of a foreign device or prosthesis in humans were eligible for this review. Inclusion and exclusion criteria were developed, tested, and refined prior to use in the review process. We did not limit our search results by year or language. See Supplementary Table 1.

### Screening

**Fig. 1:**
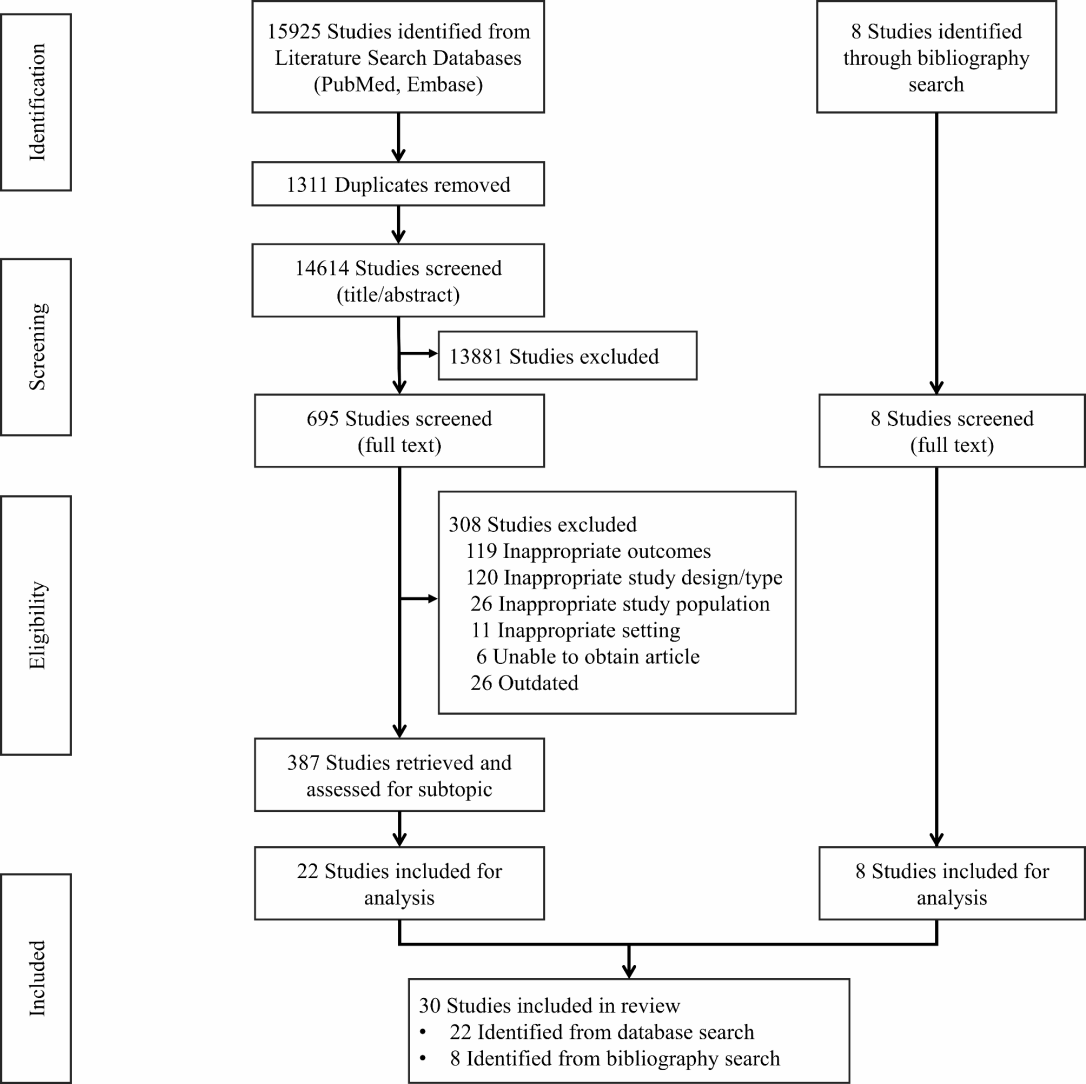
PRISMA flow diagram

Covidence (Melbourne, Australia) was used to identify and remove duplicate articles. The research team reviewed duplicate identification and removal by Covidence and made corrections as necessary. Covidence was also used for title and abstract screening by two independent reviewers. The full-text of articles included after title and abstract screening were located, and examined by two independent reviewers using the same inclusion and exclusion used for title and abstract screening. The reference lists and the list of newer articles citing the selected articles were examined using Web of Science to identify relevant articles not identified by the initial search. The gray literature was examined through Google and Google Scholar. Recommendations of subject experts were also included in the set of articles from which data was extracted. Disagreements at each stage of screening were resolved by consensus and discussion. A vote cast by a subject expert or third research team member was used as needed for final resolution. The selection process is depicted in detail in the PRISMA flow diagram (Fig. 1).

### Data extraction and analysis

Data extraction was conducted using an extraction form iteratively developed and piloted by the research team. The extraction form data fields include study type, language of the article, subject demographic information, comorbidities, history of prior malignancies, implant details (type, material, location), indications for implantation, procedures performed for implant placement, malignancy details including histology, location, and stage, time from implant to presentation, presenting symptoms, and treatment details and course. Studies were grouped and summarized by prosthesis type and associated cancer type.

## Result

The literature search identified 15,925 studies. After removal of 1,311 duplicates, 14,614 title and abstracts were screened. Full-text evaluation was conducted in 695 studies. Eight studies were identified from bibliographic search. Eventually, 30 studies, encompassing 41 cases of malignancy following orbital foreign device were included for analysis. All studies included in the evaluation comprised either case reports or case series, detailing various prosthesis types used in these cases including scleral shell, orbital implants, glaucoma implants, scleral buckles, encircling bands, and gold plates.

Scleral shells were frequently utilized in patients undergoing enucleation or evisceration for various indications, as well as in those with microphthalmia and those requiring a Gunderson flap following orbital trauma [2]. Orbital implants were primarily employed to maintain orbital volume post-enucleation, with rare exceptions where an orbital implant was inserted into the remaining scleral socket following evisceration [11]. Scleral buckles and/or encircling bands were commonly employed in retinal detachment repairs, while gold plates were inserted for the correction of lagophthalmos.

### Categorized by types of prosthesis

Among the cases categorized by type of prostheses, 35 cases were associated with scleral shell, either with or without orbital implant. One case [11] was related solely to an orbital implant placed in the scleral socket. Additionally, four cases [12–15] were linked to scleral buckle and/or encircling band use in retinal detachment repair, and one case [16] was associated with gold plate insertion.

A total of 35 scleral shell-related malignancies exhibited a mean onset time of 39.8 years (range, 4 ~ 70 years). Most cases (26 out of 29) were ocular surface squamous neoplasms (OSSN), including squamous cell carcinoma (SCC) and SCC in situ. The remaining cases included sebaceous carcinoma, marginal zone B-cell lymphoma, mucoepidermoid carcinoma, adenocarcinoma, malignant melanoma, and metastatic lung adenocarcinoma. OSSN cases primarily manifested in the tarsal, forniceal, or bulbar conjunctiva, while other malignancies occurred at various locations, all presumed to be in contact with the scleral shell. The case, which was solely related to an intrascleral orbital implant, developed intraocular pleomorphic sarcoma, filling the entire space between the sclera and the prosthesis. Among the cases with scleral shells, seven had a prior malignancy as the cause of enucleation (retinoblastoma in six cases and uveal melanoma in one), and none of these cases showed recurrence of the original malignancies.

In the context of scleral buckle and/or encircling band use, four cases developed malignancies with an average onset time of 14.8 years (range, 10 ~ 24 years) [13]. Among these cases, both SCC and synovial sarcoma were identified. Remarkably, two intraocular tumors (one SCC and one synovial sarcoma) were located adjacent to the site of buckle or band placement, despite their distinct location within the eye compared to the external positioning of the buckle/band.

One case related to gold plate developed marginal zone B-cell lymphoma after 0.7 years, presenting at the peri-implant area of the upper lid.

Detailed information is summarized in Table 1.

**Table 1:** Cases from literatures categorized by type of prosthesis

Implant	Implant material	Implant location	Implant related procedure	Cause of previous surgery	Age/Sex race	Onset time (year)	Symptom	Diagnosis	Tumor location	Reference article
**Scleral shell (+/- orbital implant)**										
Scleral shell	Artificial resin	Under eyelids	Enucleation	Recurrent RD	54/M	30	Mass	SCC	Lower palpebral conjunctiva	Croce [34]
Scleral shell	Unk	Under eyelids	Enucleation	Trauma	51/M	40	Ill-fitting prosthesis	SCC	lower conjunctiva	Endo [3]
Scleral shell	Unk	Under eyelids	Enucleation	Trauma	51/M	40	Mucopurulent discharge, ill-fitting prosthesis	SCC	Upper, lower eyelid skin Palpebral, orbital conjunctiva Parotid LN	Gaier [4]
Scleral shell	Unk	Under eyelids	Enucleation	Trauma	75/M	63	Indurated mass	SCC	Socket conjunctiva	Hsu [35]
Scleral shell	Unk	Under eyelids	Enucleation	Trauma	58/M	51	Ill-fitting prosthesis, bloody discharge	SCC	Upper bulbar conjunctiva	Nguyen [7]
Scleral shell	Unk	Under eyelids	Enucleation	Trauma	56/M	44	Ill-fitting prosthesis	SCC	Bulbar conjunctiva	Nguyen [7]
Scleral shell	Unk	Under eyelids	Enucleation	Retinoblastoma	20/M, Caucasian	13	None	OSSN	Superior tarsal, bulbar, forniceal conjunctiva	Shields [2]
Scleral shell	Unk	Under eyelids	Enucleation	Trauma	65/M	50	Mass, bloody discharge	SCC	Socket conjunctiva	Chaudhry [36]
Scleral shell	Unk	Under eyelids	Enucleation	Trauma	62/M, White	60	Mass	SCC	Superior fornix	Espana [37]
Scleral shell	Unk	Under eyelids	Enucleation	Trauma	60/M	53	Swelling, pain, ill-fitting prosthesis	SCC	Upper bulbar, palpebral conjunctiva	Jain [5]
Scleral shell	Unk	Under eyelids	Enucleation	Trauma	59/M	30	Mass, prosthesis displacement	SCC	Superior tarsal conjunctiva	McGrath [6]
Scleral shell	Unk	Under eyelids	Enucleation	Trauma	63/M	42	Mass, prosthesis displacement	SCC	Superior bulbar conjunctiva	McGrath [6]
Scleral shell	Unk	Under eyelids	Enucleation	Retinoblastoma	65/F	63	Mass, mucoid discharge	SCC in situ	Inferior tarsal conjunctiva	McGrath [6]
Scleral shell	Unk	Under eyelids	Enucleation	Trauma	73/F	10	Mass	SCC in situ	Superior tarsal conjunctiva	McGrath [6]
Scleral shell	Unk	Under eyelids	Enucleation	Trauma	62/M, Caucasian	49	Mass, discomfort, yellow discharge	SCC	Lower lid skin & palpebral conjunctiva	Whittaker [38]
Scleral shell	Unk	Under eyelids	Trauma (s/p Gunderson flap)		43/M, Caucasian	26	Mucous discharge	OSSN	Superior forniceal conjunctiva, caruncle	Shields [2]
Scleral shell	Unk	Under eyelids	Microphthalmia		60/F, Caucasian	54	Nodule	SCC	Inferior tarsal conjunctiva	Shields [2]
Scleral shell	Unk	Under eyelids	Trauma		59/M	40	Mass	SCC	Upper eyelid, palpebral conjunctiva, into orbit	Hayashi [39]
Scleral shell	Unk	Under eyelids	Enucleation	Retinoblastoma	40/M	38	Mass	SCC	Bulbar conjunctiva	Oka [40]
Scleral shell	Unk	Under eyelids	Enucleation	Retinoblastoma	26/F	25	Mass	SCC	Bulbar conjunctiva	Oka [40]
Scleral shell	Unk	Under eyelids	Enucleation	Retinoblastoma	43/M	41	Discomfort, swelling	Conjunctival carcinoma	Socket conjunctiva, parotid LN, cervical LN	Moulin [41]
Scleral shell	Unk	Under eyelids	Enucleation	Congenital glaucoma	68/F	65	Swelling, non-bloody discharge, ill-fitting prosthesis	Sebaceous carcinoma	Orbital cavity	Shibata [42]
Scleral shell	Unk	Under eyelids	Enucleation	Sympathetic ophthalmia	75/M	27	Discomfort	Sebaceous carcinoma	Orbital cavity	Tanaka [43]
Scleral shell	Unk	Under eyelids	Enucleation	Trauma	76/M	28	Ill-fitting prosthesis	Marginal zone B-cell lymphoma	Socket conjunctiva Cervical LN, BM	Dolan [44]
Scleral shell	Unk	Under eyelids	Evisceration		69/F	63	Ill-fitting prosthesis	Malignant melanoma (mixed type)	Anterior, adjacent to eviscerated globe (remnant uveal tissue)	Kita [45]
Scleral shell	Unk	Under eyelids	Enucleation	Intraorbital neuropathy (Complication after alcohol injection)	59/M	12	Mass, prosthesis displacement	Malignant melanoma (spindle cell type)	Orbital cavity (intraconal & extraconal compartment, contact with scleral shell)	Ferreira [46]
Scleral shell, orbital implant	Unk	Under eyelids, Anophthalmic socket	Enucleation	Trauma	80/M, Caucasian	70	Mass	SCC in situ	Inferior conjunctival fornix	Bonavolontà [47]
Scleral shell, orbital implant	Unk	Under eyelids, Anophthalmic socket	Enucleation	Retinoblastoma	38/M	36	Itchy, painless, bloody nodule	SCC	Inferior conjunctival fornix	Bonavolontà [47]
Scleral shell, orbital implant	Hydroxyapatite (implant)	Under eyelids, Anophthalmic socket	Enucleation	Trauma	56/M	10	Painless	SCC	Inferior conjunctival fornix	Bonavolontà [47]
Scleral shell, orbital implant	Unk	Under eyelids, Anophthalmic socket	Enucleation	Trauma	69/M	49	Mass, serosanguineous discharge, discomfort	SCC	Superior tarsopalpebral conjunctiva	Campanella [48]
Scleral shell, orbital implant	Gold (implant)	Under eyelids, Anophthalmic socket	Enucleation	Uveal melanoma	77/M	43	Discomfort, swelling	SCC	Tarsopalpebral conjunctiva, superior conjunctival fornix, upper lid	Campanella [48]
Scleral shell, orbital implant	Unk	Under eyelids, Anophthalmic socket	Enucleation	Trauma	58/M	32	Ill-fitting prosthesis, mucoid discharge	SCC in situ	Superior fornix	Barrett [8]
Scleral shell, orbital implant	Unk	Under eyelids, Anophthalmic socket	Enucleation	Trauma	58/M, Caucasian	52	Ill-fitting prosthesis	Mucoepidermoid carcinoma	Socket conjunctiva, lower superior conjunctival fornix, palpebral chord	Bonavolontà [47]
Scleral shell, orbital implant	Unk	Under eyelids, Anophthalmic socket	Enucleation	Refractory glaucoma in non-functional traumatic eye	70/F	4	Pain, swelling, induration	Anaplastic, sarcomatoid adenocarcinoma	External lateral orbit (circumscribing scleral shell)	Liard [49]
Scleral shell, orbital implant	Unk	Under eyelids, Anophthalmic socket	Enucleation	Trauma	69/M	40	Ill-fitting prosthesis	Metastatic lung adenocarcinoma	Socket	Bussières [50]
**Orbital implant only**										
orbital implant	Silicone	Scleral pocket	Evisceration	Trauma	59/M, Caucasian	8	Pain, conjunctival hemorrhage	Undifferentiated pleomorphic sarcoma	Scleral socket (entire space between prosthesis, conjunctival invasion through previous scleral incision)	Piscitelli [11]
**Scleral buckle and/or encircling band**										
Scleral buckle	Unk	Scleral surface	RD repair	RD	29/M	12	Pain, foreign body sensation, redness	Synovial sarcoma	Intraocular (vitreous chamber, retina, optic nerve head)	Richards [15]
Scleral buckle, encircling band	Silicone	Scleral surface	RD repair	RD	48/M, Asian	24	Visual field defect	Synovial sarcoma	Intraocular (behind iris) Ciliary body invasion (beneath the site of silicone band)	Ito [14]
1) Scleral buckle, encircling band, 2) IOL	Silicone	Scleral surface	RD repair IOL for cataract	1) RD 2) Cataract	81/M, Caucasian	10	Discharge, pain, red eye	SCC	Superonasal conjunctival fornix (surrounding the site of scleral exoplant)	Lee [12]
Encircling band	Silicone	Scleral surface	RD repair	RD	76/M	13	Reduced vision (hand movement)	SCC	Choroid & orbit (at the site of encircling band)	Loffler [13]
**Gold plate**										
Gold plate	Gold	Upper lid	Gold plate insertion	Lagophthalmos	72/M	0.7	Erythema, edema	Marginal zone B cell lymphoma	Upper lid (peri-implant)	Di Nisio [16]

RD, retinal detachment; SCC, squamous cell carcinoma; OSSN, ocular surface squamous neoplasia; bx, biopsy; CTx, chemotherapy; RTx, radiation therapy; LN, lymph node; BM, bone marrow

### Categorized by cancer type

Among the cases categorized by cancer type, OSSN was predominant, accounting for 29 cases with a mean age of 57.9 years (range: 20–81 years). These cases were predominantly associated with scleral shell, either with or without orbital implant, or retinal detachment repair involving an encircling band and/or scleral buckle. OSSN tumors typically localized in the tarsal, forniceal, or bulbar conjunctiva, except for one case which occurred in association with encircling band. In this case, the tumor was located at choroid and orbit, at the site of encircling band, with no connection to conjunctiva.

Additionally, various other malignancies were observed, including mucoepidermoid carcinoma, pleomorphic sarcoma, synovial sarcoma, marginal zone B-cell lymphoma, sebaceous carcinoma, malignant melanoma, adenocarcinoma, or metastatic lung adenocarcinoma. Three sarcoma cases were located within the scleral socket, one case occurring after intrascleral orbital implant and the other two associated with scleral buckle and/or encircling band related to retinal detachment repair. Both sebaceous carcinoma cases occurred in orbital cavity of the anophthalmic socket. Two malignant melanoma cases occurred at anterior eviscerated globe and within orbital cavity with close contact to scleral shell.

Each malignancy exhibited unique characteristics and demographics, with some cases showing metastasis or local invasion. Two cases of SCC and one case of sebaceous carcinoma following scleral shell placement demonstrated regional lymph node metastasis. Additionally, the case of marginal zone B-cell lymphoma, also following scleral shell placement, showed cervical lymph node and bone marrow involvement.

Detailed demographic data, details of prosthesis, symptoms, onset time, tumor location, treatment, and other associated factors for each case are summarized in Table 2 for reference. Mass-like lesion was the most common presentation, regardless of cancer type. Ill-fitting prostheses and discharge were also common symptoms.

**Table 2:** Cases from literatures categorized by type of cancer

Diagnosis	Implant	Age/Sex race	onset time (year)	Symptom	Tumor location (initial presentation)	Treatment	Follow-up	Other factors	Reference article
**OSSN**									
OSSN	Scleral shell	43/M, Caucasian	26	Mucous discharge	Superior forniceal conjunctiva, caruncle	Topical, intralesional IFNa2b	69mo	Chronic use of topical steroids	Shields [2]
SCC	Scleral shell	60/F, Caucasian	54	Nodule	Inferior tarsal conjunctiva	Topical, intralesional IFNa2b	3mo	Chronic use of topical steroids	Shields [2]
SCC	Scleral shell	54/M	30	Mass	Lower palpebral conjunctiva	Excision	6mo		Croce [34]
SCC	Scleral shell	51/M	40	Ill-fitting prosthesis	Lower conjunctiva Submandibular LN	Exenteration, CTx, RTx	15mo		Endo [3]
SCC	Scleral shell	51/M	40	Mucopurulent discharge, ill-fitting prosthesis	Upper, lower eyelid skin Palpebral, orbital conjunctiva Parotid LN	Exenteration, XRT	6mo	HPV 16 positive	Gaier [4]
SCC	Scleral shell	75/M	63	Indurated mass	Socket conjunctiva	Exenteration, XRT	24mo	Chronic irritation	Hsu [35]
SCC	Scleral shell	58/M	51	Ill-fitting prosthesis, bloody discharge	Upper bulbar conjunctiva	Exenteration	12mo		Nguyen [7]
SCC	Scleral shell	56/M	44	Ill-fitting prosthesis	Bulbar conjunctiva	Exenteration	24mo	Active smoker	Nguyen [7]
OSSN	Scleral shell	20/M, Caucasian	13	None	Superior tarsal, bulbar, forniceal conjunctiva	Topical and intralesional IFNa2b	9mo	Prior malignancy (retinoblastoma) Chronic irritation	Shields [2]
SCC	Scleral shell	65/M	50	Mass, bloody discharge	Socket conjunctiva	Excision, RTx	36mo	Chronic irritation	Chaudhry [36]
SCC	Scleral shell	62/M, White	60	Mass	Superior fornix	Excision, Mitomycin-C, cryotherapy, exenteration	1mo		Espana [37]
SCC	Scleral shell	60/M	53	Swelling, pain, ill-fitting prosthesis	Upper bulbar, palpebral conjunctiva	Exenteration	Unk	Ex-smoker	Jain [5]
SCC	Scleral shell	59/M	30	Mass, prosthesis displacement	Superior tarsal conjunctiva	Cryotherapy, exenteration	36mo	HPV 16 positive, Active smoker	McGrath [6]
SCC	Scleral shell	63/M	42	Mass, prosthesis displacement	Superior bulbar conjunctiva	Incisional bx, cryotherapy, intralesional IFNa2a, exenteration	Unk	HPV 16 positive	McGrath [6]
SCC in situ	Scleral shell	65/F	63	Mass, mucoid discharge	Inferior tarsal conjunctiva	Incisional bx, cryotherapy, intralesional IFNa2a	32mo	Prior malignancy (retinoblastoma) HPV16 negative	McGrath [6]
SCC in situ	Scleral shell	73/F	10	Mass	Superior tarsal conjunctiva	Incisional bx, mitomycin-C	6mo	HPV 16 positive	McGrath [6]
SCC	Scleral shell	62/M, Caucasian	49	Mass, discomfort, yellow discharge	Lower lid skin & palpebral conjunctiva	Excision	7mo	Active smoker, well-fitted prosthesis	Whittaker [38]
SCC	Scleral shell	40/M	38	Mass	Bulbar conjunctiva	Excision	Recur after 2yr	Prior malignancy (retinoblastoma)	Oka [40]
SCC	Scleral shell	26/F	25	Mass	Bulbar conjunctiva	Excision	Unk	Prior malignancy (retinoblastoma)	Oka [40]
SCC	Scleral shell	59/M	40	mass	Upper eyelid, palpebral conjunctiva, into orbit	Exenteration	5yr	HPV 6,11,16,18 negative	Hayashi [39]
SCC in situ	Scleral shell, orbital implant	80/M, Caucasian	70	Mass	Inferior conjunctival fornix	Excision	1yr	Well managed prosthesis	Bonavolontà [47]
SCC	Scleral shell, orbital implant	38/M	36	Itchy, painless, bloody nodule	Inferior conjunctival fornix	Excision	2yr	Prior malignancy (retinoblastoma) Well managed prosthesis	Bonavolontà [47]
SCC	Scleral shell, orbital implant	56/M	10	Painless	Inferior conjunctival fornix	Excision	19mo	Well managed prosthesis	Bonavolontà [47]
SCC	Scleral shell, orbital implant	69/M	49	Mass, serosanguineous discharge, discomfort	Superior tarsopalpebral conjunctiva	Exenteration	Unk	h/o poorly fitting prosthesis, ex-smoker	Campanella [48]
SCC	Scleral shell, orbital implant	77/M	43	Discomfort, swelling	Tarsopalpebral conjunctiva, superior conjunctival fornix, upper lid	Exenteration	Unk	Prior malignancy (uveal melanoma)	Campanella [48]
SCC in situ	Scleral shell, orbital implant	58/M	32	Ill-fitting prosthesis, mucoid discharge	Superior conjunctival fornix	Topical mitomycin-C	10mo	Original prosthesis w/o maintenance	Barrett [8]
SCC	1) Scleral buckle, encircling band 2) IOL	81/M, Caucasian	10	Discharge, pain, red eye	Superonasal conjunctival fornix (surrounding the site of scleral exoplant)	Exenteration	18mo	h/o lymphoma Cataract op. c post. Chamber IOL	Lee [12]
SCC	Encircling band	76/M	13	Reduced vision (hand movement)	Choroid & orbit (at the site of encircling band)	Exenteration	17mo		Loffler [13]
Conjunctival carcinoma	Sclereal shell	43/M, Caucasian	41	Discomfort, swelling	Socket conjunctiva, parotid LN, cervical LN	Proton therapy, parotidectomy, cervical LN dissection, RTx, CTx	6mo	Prior malignancy (retinoblastoma) Smoker	Moulin [41]
**Sarcoma**									
Undifferentiated pleomorphic sarcoma	Orbital implant * (inside scleral socket)	59/M, Caucasian	8	Pain, conjunctival hemorrhage	Scleral socket (entire space between prosthesis, conjunctival invasion through previous scleral incision)	Exenteration, RTx	Unk		Piscitelli [11]
Synovial sarcoma	Scleral buckle	29/M	12	Pain, foreign body sensation, redness	Intraocular (vitreous chamber, retina, optic nerve head)	Enucleation	Unk		Richards [15]
Synovial sarcoma	Scleral buckle, encircling band	48/M, Asian	24	Visual field defect	Intraocular (just behind iris) Ciliary body invasion (beneath the site of silicone band)	Enucleation	6mo		Ito [14]
**Lymphoma**									
Marginal zone B cell lymphoma	Gold plate	72/M	0.7	Erythema, edema	Upper lid (peri-implant)	Incisional bx, RTx, gold plate removal	3yr		Di Nisio [16]
Marginal zone B-cell lymphoma	Scleral shell	76/M	28	Ill-fitting prosthesis	Orbital cavity Cervical LN, BM	CTx, RTx	Unk		Dolan [44]
**Others**									
Mucoepidermoid carcinoma	Scleral shell, orbital implant	58/M, Caucasian	52	Ill-fitting prosthesis	Orbital cavity, lower superior conjunctival fornix, palpebral chord	Exenteration	Recur after 10mo	Well managed prosthesis	Bonavolontà [47]
Sebaceous carcinoma	Scleral shell	68/F	65	Swelling, non-bloody discharge, ill-fitting prosthesis	Orbital cavity	Exenteration	10mo	Original prosthesis w/o maintenance	Shibata [42]
Sebaceous carcinoma	Scleral shell	75/M	27	Discomfort	Orbital cavity Parotid LN	Exenteration	2.5yr		Tanaka [43]
Malignant melanoma (mixed type)	Scleral shell	69/F	63	Ill-fitting prosthesis	Anterior, adjacent to eviscerated globe (remnant uveal tissue)	Exenteration	Unk		Kita [45]
Malignang melanoma (spindle cell type)	Scleral shell	59/M	12	Mass, prosthesis displacement	Orbital cavity (intraconal & extraconal compartment, contact with scleral shell)	Unk	Unk		Ferreira [46]
Anaplastic & sarcomatoid adenocarcinoma	Scleral shell, orbital implant,	70/F	4	Pain, swelling, induration	External lateral orbit (circumscribing prosthesis)	Exenteration, RTx	Unk		Liard [49]
Metastatic lung adenocarcinoma	Scleral shell, orbital implant	69/M	40	Ill-fitting prosthesis	Anophthalmic socket	Excision	DOD 1mo	ex-smoker	Bussières [50]

RD, retinal detachment; SCC, squamous cell carcinoma; OSSN, ocular surface squamous neoplasia; bx, biopsy; CTx, chemotherapy; RTx, radiation therapy; LN, lymph node; BM, bone marrow; DOD, die of disease

## Discussion

Ocular malignancies, though relatively rare, pose significant clinical challenges, with annual incidence rates ranging from 0.1 to 1.3 per 100,000 individuals, exhibiting variations based on factors such as race, nationality, and sex [17–19]. Established risk factors for ocular cancers include advanced age, ultraviolet (UV) irradiation, race/ethnicity, and genetic predispositions [20]. Immunosuppressive status, such as HIV infection, or HPV has been suggested to be associated with OSSN. However, beyond commonly known cancers like uveal melanoma and OSSN, specific risk factors for other ocular malignancies remain poorly understood.

While various prostheses are utilized in ocular procedures, their potential association with carcinogenesis is inadequately studied, with some existing studies reporting malignancies linked to ocular prostheses. Nonetheless, comprehensive research on ocular cancer development in relation to foreign device is lacking. Therefore, our study aimed to address this gap by reviewing the types of malignancies occurring after various orbital foreign devices and their characteristics.

A recent study has suggested chronic inflammation as a potential risk factor for conjunctival SCC [21]. While continuous irritation resulting in repeated ulceration and inadequate healing is well accepted as a carcinogenic mechanism for skin SCC [22], conjunctival SCC is conjectured to share a similar mechanism [23]. It has been reported that squamous metaplasia is observed even in conjunctiva of healthy-appearing anophthalmic sockets [24]. Additionally, chronic inflammation caused by conditions such as dry eye has been reported to induce squamous metaplasia [25, 26]. It is also suggested that common mechanisms regulate the initiation of SCC regardless of tissue type across the body [27]. 

In general, contribution of inflammation to cancer development is well understood [28]. Considering the impact of chronic irritation caused by scleral shell on carcinogenesis of OSSN, the role of inflammation in cancer development becomes significant. Furthermore, beyond OSSN, ocular adnexal lymphoma is understood as an extension of lymphoproliferative disorders [29], suggesting a potential role for foreign body-induced inflammation in the development of other rare ocular malignancies. While it is possible that the development of malignancy may be independent of the prosthesis and associated with other risk factors or underlying conditions, the potential association between chronic inflammation caused by orbital foreign devices and carcinogenesis should not be overlooked.

Our study also uncovered disparities between the reported frequencies of malignancies following various prosthesis-related procedures and the frequencies of the procedures themselves [30, 31]. For instance, despite the rarity of evisceration and enucleation procedures, the placement of scleral shell post-evisceration or enucleation was frequently associated with subsequent malignancies. This discrepancy may be attributed to differing carcinogenic susceptibilities among tissues in contact with prostheses at various locations within the eye. Tissues with a higher rate of cell turnover, such as conjunctiva, may be more prone to inflammation-induced carcinogenesis compared to tissues with lower rates of turnover. Furthermore, the concept of ocular immune privilege may have contributed to these discrepant frequencies [32, 33]. Prostheses placed in immune-privileged sites, such as intraocular lens, may have lower likelihood of inducing chronic inflammation, thus reducing the risk of inflammation-induced carcinogenesis.

Interestingly, some malignancies developed in spaces different from the prostheses placement following retinal detachment repair. Although encircling band or scleral buckle are placed outside the sclera, tumors occurred intraocularly, at the site of the band or buckle. This raises the possibility of cytokine and chemokine diffusion from the prosthesis site, potentially influencing nearby tissues. The prosthesis-related inflammatory microenvironment may not only affect the location of the prosthesis but also adjacent areas through the release of cytokine or chemokine.

Our study identified a range of malignancies occurring after various orbital foreign device-related procedures. Although these occurrences are exceedingly rare, our findings underscore the importance of postoperative surveillance for clinicians. Patients with risk factors, such as HPV-16 infection, which may contribute to SCC development, should be particularly vigilant about this risk and ensure regular follow-up exams. By raising awareness of the potential for cancer development following such procedures, clinicians can prioritize vigilant monitoring, leading to early detection and timely intervention.

While our scoping review provides valuable insights, it is not without limitations. Variability in reported case details, including specific materials of prostheses and associated risk factors, hampers comprehensive interpretation. Most articles lack information regarding materials used, limiting our understanding of detailed carcinogenic mechanisms. Nevertheless, we posit that prosthesis-related inflammatory reactions, irrespective of material type, play a pivotal role in carcinogenesis. Although detailed clinical information is often missing from the literatures, most malignancies occurred adjacent to the foreign device. Additionally, as is inherent in scoping reviews, despite our comprehensive search strategy, there remains the possibility that some relevant cases that may not have been captured in this study.

It is important to note that this scoping literature review is not an epidemiologic study and cannot accurately evaluate the incidence of malignancies in patients with orbital foreign devices and is insufficient to be supporting evidence for causal relationship. Nonetheless, we believe our work is of value as an initial step in acknowledging the existence of malignancies developing after orbital foreign device-related procedures, as our study suggests a potential association between various types of orbital foreign devices and malignancies. While we hypothesize the incidence to be rather rare, we believe that acknowledgement of the Oppenheimer effect and the potential for malignancy to develop adjacent to a foreign device is worthy of further investigation. Future studies, including well-designed epidemiologic studies and scientific experiments, are warranted to elucidate the causal relationship.

## Electronic supplementary material

Below is the link to the electronic supplementary material.

Supplementary Material 1

Supplementary Material 2

## Data Availability

No datasets were generated or analysed during the current study.

## Abbreviations

BIA-ALCL: Breast implant-associated anaplastic large cell lymphoma
IRB: Institutional Review Board
OSSN: Ocular surface squamous neoplasms
SCC: Squamous cell carcinoma
UV: Ultraviolet
