# Supplementary material for: Investigating orbital foreign device-associated malignancies: a scoping review
Source: BMC Cancer. 2025 Jan 28;25:167. doi: 10.1186/s12885-024-13422-z (PMC11776229; doi:10.1186/s12885-024-13422-z)
Supplement: Supplementary file 1 — Supplementary Material 1 [file 12885_2024_13422_MOESM1_ESM.docx]

**Supplementary Table 1.**

| **Inclusion** | **Exclusion** |
| --- | --- |
| Implantation or use of a foreign device or prosthesis | Non-human subjects |
| Human subjects | Articles reporting outcomes related to benign masses (e.g., cysts, neuromas, papillomas, fibromas, and lipomas) |
| Studies examining external prostheses, e.g., pessaries, dentures, and indwelling catheters | No language restrictions |
| De novo malignancies at site of implantation  OR  De novo malignancies that were systemic or distant from the prosthesis occurring after prosthesis implantation/use  OR  Metastases of any tumor that were found adjacent to or near a prosthesis. | No data restrictions |
| Clinical studies, cohort studies, case-control studies, case reports, case series, and conference abstracts | Articles without original data, such as meta-analyses, systematic reviews, practice guidelines, book chapters, narrative reviews, news, editorials, or comments |
